# Neuroinflammation driven by Human Immunodeficiency Virus-1 (HIV-1) directs the expression of long noncoding RNA RP11–677M14.2 resulting in dysregulation of Neurogranin in vivo and in vitro

**DOI:** 10.21203/rs.3.rs-3810214/v1

**Published:** 2024-01-03

**Authors:** Roberta S. Dos Reis, Marc C.E. Wagner, Savannah McKenna, Velpandi Ayyavoo

**Affiliations:** University of Pittsburgh; University of Pittsburgh; University of Pittsburgh; University of Pittsburgh

## Abstract

Neuroinflammation and synaptodendritic damage represent the pathological hallmarks of HIV-1 associated cognitive disorders (HAND). The post-synaptic protein neurogranin (Nrgn) is significantly reduced in the frontal cortex of postmortem brains from people with HIV (PWH) and it is associated with inflammatory factors released by infected microglia/macrophages. However, the mechanism involved in synaptic loss have yet to be elucidated. In this study, we characterized a newly identified long non-coding RNA (lncRNA) transcript (RP11–677M14.2), which is antisense to the NRGN locus and is highly expressed in the frontal cortex of HIV-1 individuals. Further analysis indicates an inverse correlation between the expression of RP11–677M14.2 RNA and Nrgn mRNA. Additionally, the Nrgn-lncRNA axis is dysregulated in neurons exposed to HIV-1 infected microglia conditioned medium enriched with IL-1b. Moreover, *in vitro* overexpression of this lncRNA impact Nrgn expression at both mRNA and protein levels. Finally, we modeled the Nrgn-lncRNA dysregulation within an HIV-1-induced neuroinflammatory environment using brain organoids, thereby corroborating our *in vivo* and *in vitro* findings. Together, our study implicates a plausible role for lncRNA RP11–677M14.2 in modulating Nrgn expression that might serve as the mechanistic link between Nrgn loss and cognitive dysfunction in HAND, thus shedding new light on the mechanisms underlying synaptodendritic damage.

## INTRODUCTION

HIV-1 associated neurocognitive disorder (HAND) remains as one of the most prevalent HIV-1 related comorbidities, despite systemic viral suppression [1]. The infectious microenvironment established upon viral entry in brain provokes changes in neuronal structure and function, as dendritic simplification and synaptic loss, particularly in the frontal cortex [2, 3]. These structural and functional changes are paired with deficits in memory and cognition, increasing the risk of poorer health outcomes in people with HIV-1 (PWH). Currently, there is no treatment that can prevent or restore such damage. Therefore, it is critically important to understand the molecular and cellular mechanisms behind neuronal dysregulation and cognitive impairment upon HIV-1 infection of the human brain.

We have previously reported that Neurogranin (Nrgn), a post-synaptic protein present in dendrites is directly associated with cognitive processes such as learning, memory and executive functions, are dysregulated in the brain of PWH [4, 5]. Consequently, loss of Nrgn contributes to establishment of the synaptodendritic damage, the neuropathological hallmark underlying the cognitive deficits observed in HIV-associated neurocognitive disorders (HAND) [4, 6]. However, very little is known regarding Nrgn expression, and the mechanism(s) regulating HIV-1 induced Nrgn dysregulation.

Non-coding RNAs (ncRNAs) contribute to gene expression through modulation of transcriptional and post-transcriptional processes [7–9]. The long non coding RNAs (lncRNAs) can bind to several proteins, DNAs and RNAs to form functional complexes to regulate gene expression from epigenetic modifications, transcription to RNA processing, transport and translation [9, 10]. In recent years, thousands of lncRNAs have been annotated in human genome and the number of reports suggesting their functionality and implications to physiological and pathological processes are rapidly increasing. Therefore, a growing number of lncRNAs have also been reported to be altered in response to viral infections [5, 11–14]. Nevertheless, only few long ncRNAs have been functionally characterized to date and have been found to be manipulated by HIV-1 [15–17].

In this study we have identified a lncRNA transcript (RP11–677M14.2) localized in the antisense strand in NRGN locus that has not been functionally characterized. A number of natural antisense transcripts have been recently investigated and characterized as regulators of their sense mRNA expression [18–21]. Moreover, compared to intergenic and intronic localized lncRNAs, antisense lncRNAs are more stable [22], which might be good indicators for their potential biological functions. Therefore, we hypothesize that RP11–677M14.2 antisense lncRNA might regulate Nrgn mRNA expression in neurons.

Here, we report the characterization of the RP11–677M14.2 antisense lncRNA, with a focus on its physiological relevance to NRGN and HIV-1 neuropathology. We found that RP11–677M14.2 transcript is significantly enriched in frontal cortex tissue of PWH. Furthermore, higher RP11–677M14.2 expression in frontal cortex is directly correlated with lower Nrgn mRNA expression. Accordingly, overexpression of this lncRNA in neurons resulted in reduced Nrgn expression through mechanisms other than physically interacting with Nrgn mRNA. Furthermore, two-dimensional, or three-dimensional neuronal culture treatment with conditioned media from HIV-infected macrophages/microglia induced the upregulation of the lncRNA and subsequent downregulation of Nrgn mRNA. Based on these observations, we identified a new sense and antisense regulatory axis that might represent an important missing link between inflammation and synaptic dysregulation in HIV-1 neuropathogenesis.

## METHODS

### Study cohort

Tissue samples (frontal cortex) from people with HIV-1 with and without cognitive impairment were obtained from National NeuroAIDS Tissue Consortium (NNTC) and Multicenter AIDS Cohort study (MACS). Frontal cortex samples from people without HIV-1 (HIV-1 seronegative), neurocognitive normal, age and sex matched individuals were obtained from NIH Neurobiobank (University of Miami Brain Endowment Brank) and used as control. Diagnosis of HAND was based on the clinical classification redefined in 2007 [23]. The use of postmortem tissue was reviewed and approved by Committee for Oversight of Research and Clinical Training Involving Decedents (CORID) of the University of Pittsburgh. Summary of characteristics and demographics information is listed in **Supplementary tables 1 and 2, Additional file 2.** All frozen tissue samples were preserved frozen at − 80°C until required. FFPE slides were stored at room temperature until required. Blood samples from healthy adults PBMCs were obtained from Red Cross Blood bank.

### Cells

HEK293T and U87MG (NIH AIDS Reagent program), SH-SY5Y (ATCC) cells were grown in DMEM supplemented with 10% FCS, 1% glutamine and 1% penicillin-streptomycin. We induced differentiation of SH-SY5Y cells by adding 10 mM all-trans-retinoic acid (RA) to the growth medium 24 hours post plating, and RA-containing growth medium was replaced every day for 7 consecutive days. Primary adult human microglia were a gift from Dr. Changiz Geula from Northwestern University. Briefly, microglia were isolated from the prefrontal cortex of a 71-year-old Caucasian male (*postmortem* interval of 31 hours). Brain tissue from this patient was obtained from Northwestern University Alzheimer’s Disease Center Brain Bank (AG13854). Culture was maintained as previously published [24]. Briefly, cells were seeded in PDL-coated plates and kept in complete microglia medium (ScienCell Research Laboratories). Experiments were conducted with passages between 8 to 10. All cell lines were kept in a humidified incubator at 37°C and 5% CO_2_.

### Isolation of CD14 + monocytes and differentiation to macrophages (MDM)

Monocytes-derived macrophages (MDMs) were generated from normal peripheral blood mononuclear cells (PBMC). PBMCs from healthy donor were isolated by Ficoll-Hypaque gradient centrifugation. CD14 + monocytes were purified by positive selection using anti-CD14 monoclonal antibody-coated magnetic microbeads (Miltenyi Biotech, Auburn, CA) and differentiated in presence of 1 pg/ml M-CSF and 1 × 10^6^ IU/ml GM-CSF (R&D Systems) as described previously [25]. Half the volume of media was replaced every third day with fresh media containing GM-CSF and M-CSF for 7–8 days to differentiate them into MDMs.

### Virus production

HIV-1 viruses were generated using the neurotropic proviral construct pNL43-YU2 Env-eGFP. HEK293T cells (2×10^6^) were transfected with 3.5mg of proviral construct and 1.5 mg of vesicular stomatitis virus G (VSV-G) -Envelope expression plasmid using 15mL PolyJet^™^ transfection reagent (SignaGen Laboratories). The transfection mixture was gently vortexed and incubated for 20 min at room temperature to allow the formation of transfection complexes. The transfection mixture was then added dropwise to the cells and incubated at 37°C for 16 hr. The medium-containing transfection mixture was replaced using fresh complete medium, and after another 48 hour the supernatant containing viruses was removed, spun at 3000g for 10 min and filtered to remove cell debris. Virus was collected by ultracentrifugation for 60 min at 20,000 rpm (4°C) and stored at −80°C until further use. Viruses were titrated using U87MG CD4 + CCR5 + permissive cells to determine the infectivity as infectious units/ml. Adult primary microglia and MDMs were infected with HIV-1 at a multiplicity of infection of 0.5 as described before[4]. Mock infection was performed using equal amount of HEK293T supernatant. Microglia supernatants were collected 3 to 14 days post infection and MDM supernatants were collected 8 to 12 days post infection[4]. Supernatants were treated with complete protease inhibitors (Roche) and stored at −80°C until use.

### Plasmids construction and cell transfection

The cDNA encoding full length RP11–677M14.2 was PCR-amplified from undifferentiated SH-SY5Y (ASNrgnHindIII_Fw 5’-CGAAGCTTCTTTCAGTACCAGGATTCTTTGGG-3’; ASNrgnXhoI_Rev 5’-CGCTCGAGAGGTACGTAATAGCTTTATTTTGGGG-3’) and subcloned into pcDNA3.1 vector (Invitrogen). The empty pcDNA3.1 vector was used as the control. All plasmids were isolated using GeneJET Plasmid Maxiprep kit (ThermoFisher) and the specific clones were confirmed by DNA sequencing of at least 5 colonies. HEK293T and SH-SY5Y (500.000 cells) were transfected with 1mg of proviral construct using 3 mL PolyJet^™^ transfection reagent (SignaGen Laboratories) per manufacturer’s instructions. The assays were conducted 24–48 hrs after transfection.

### Total RNA extraction and quantitative real time PCR

RNA was isolated from tissue and cells using the MirVana kit (ThermoFisher) per manufacturer’s recommendations. The concentration and purity of the RNA were measured by a NanoDrop 1000 spectrophotometer (Thermo Fisher Scientific). Purity was checked by the ratio of the OD_260_/OD_280_ and OD_260_/OD_230_. The RNA was treated with DNase using a DNA-free Turbo DNase kit (Ambion). cDNA was prepared from 1 μg of total RNA using a high-capacity cDNA reverse transcription kit (ThermoFisher) in 20 μL total volume reaction. Quantitative real time PCR was performed using Taqman Universal PCR master mix (ThermoFisher) and the appropriate Taqman assays (ThermoFisher) or primers (**Supplementary table 3, Additional file 2**) with 2 μL of the RT reaction mixture. Assays were conducted on Thermo ABI ViiA7 real time PCR system in the following cycling conditions: activation of Taq DNA polymerase at 95°C for 10 min, followed by 45 cycles of amplification at 95°C for 15 s and 60°C for 1 min. Results were normalized to the expression of Ribosomal Protein Lateral Stalk Subunit P0 (RPLP0) (**Supplementary table 3, Additional file 2**).

### Subcellular fractionation

The separation of nuclear and cytosolic fractions was performed using a PARIS Kit (Life Technologies) according to the manufacturer’s instructions. RNA extraction of the fractions was performed as previously described in methods and compared to total cell RNA extracts using GAPDH and *Malat 1* as cytoplasmic and nuclear controls respectively (**Supplementary table 3, Additional file 2**).

### RNA Hybridization chain reaction - Fluorescence in situ hybridization (RNA HCR-FISH)

Multiple HCR-FISH probes were designed by Molecular Instruments to hybridize to various sequences along the Nrgn mRNA and RP11–677M14.2 transcript. For simultaneous amplification in different color channels, HCR initiators were appended to tiled sequences via a 2-base spacer (AA) creating even- and odd-tiled sequences probes. For culture cells, we used 4.8 pmol of each of the pooled HCR RNA FISH probes sets per 300 mL of hybridization buffer (Molecular Instruments) per coverslip, according to manufacturer’ protocol. Hybridization of initiation probes was carried out in humidified chamber overnight at 37°C. Before the amplification step, we snap-cooled 6 μL per well of individual 3 μM HCR hairpins amplifiers (Molecular Instruments) conjugated to Alexa Fluor 647 (Alexa 647) or Alexa Fluor 488 (Alexa 488) in separate PCR tubes by heating at 95°C for 90 s and immediately transferring it to room temperature for 30 min protected from light. Next, we pooled the hairpins in 300 μL of amplification buffer to a final concentration of 60 nM each and added to samples after washing them with wash buffer (Molecular Instruments). The coverslips were incubated at room temperature overnight protected from light. Post hairpin amplification, we washed the samples 5 times for 5 min with 5× SSCT, added 100 μL of 100 ng/mL of DAPI (Invitrogen) solution in PBS for 1 min. Samples were mounted on slides with gelvatol and we proceeded to image them.

For co-detection of protein and RNA with HCR RNA FISH on formalin-fixed paraffin-embedded (FFPR) tissues, we first deparaffinized the sections on slides with serial grades of ethanol rehydration washes. We washed the slides in nuclease-free water for 3 min, and then performed antigen retrieval by immersing the slides in a heated solution (of 10 mM sodium citrate (pH 6) for 15 min at 95 °C. After antigen retrieval, we rinsed the cooled slides with 1x PBS 0.1% Tween 20 and immediately proceeded to the protein detection stage, according to manufacturer’s instructions (Molecular Instruments). Briefly, sections were permeabilized with 0.1% Triton-X-PBS for 15 min followed by blocking with 2% BSA for 1 h. Next, sections were incubated with primary antibodies against human Nrgn (1:1000-kindly donated by Drs. Everett and Yang from John Hopkins University) overnight at 4°C. Tissue sections were washed five times with 0.5% BSA in PBS and were further incubated with goat anti-rabbit-Cy3. Samples were post-fixed with 4% formaldehyde for 10 min, washed in PBST and 5x SSCT and we immediately proceeded to the hybridization stage. Each tissue sections were incubated with 1.6 pmol of lncRNA probe set in 100 μL of hybridization buffer overnight in the 37°C humidified chamber. After the primary probe hybridization, we washed the samples by immersion sequentially in 75% wash buffer (Molecular Instruments) plus 25% 5× SSCT (5× SSC + 0.1% Tween 20) solution, 50% wash buffer plus 50% 5× SSCT solution, 25% wash buffer plus 75% 5× SSCT solution, and 100% 5× SSCT for 15 min each at 37°C. We then washed the samples in 5× SSCT at room temperature for 5 min and incubated the samples at room temperature for 30 min in an amplification buffer (Molecular Instruments). During this incubation, we snap-cooled, by heating at 95°C for 90 s in separate PCR tubes, 2 μL per slide of individual 3 μM HCR hairpins (Molecular Instruments) conjugated to Alexa 647 and immediately transferred the samples to room temperature to cool for 30 min protected from light. After, we pooled the hairpins in 100 μL of amplification buffer per slide to a final concentration of 60 nM each. We added the hairpin solution to samples, placed a glass coverslip on top, and then incubated samples at room temperature overnight protected from light. After hairpin amplification, we washed samples 1 × 5 min in 5× SSCT, 2 × 15 min in 5× SSCT, and then 1 × 5 min with 5× SSCT again. We then stained nuclei by adding 100 μL of 5× SSCT containing 100 ng/mL of DAPI to each slide for 5 min at room temperature, added mounting media and coverslip, and then proceeded to image the samples.

### Generation of brain organoids and RNA extraction

To generate brain organoids incorporated with mock and HIV-1-infected microglia we followed the protocol previously established [26]. Briefly, microglia (both mock and HIV-infected) were detached from the 2D flasks and incubated with two-week old human brain organoids (hBORGs), previously rinsed with PBS, as the ratio of 1 microglia cell to 20 NPCs. Microglia and hBORGs were incubated without agitation for 24h to allow attachment of microglia to the hBORG surface. The MG-hBORGs were then carefully transferred to a new plate with fresh differentiation media to remove unattached MGs and were maintained in culture in differentiation media for an additional 30 days. To extract RNA from MG-hBORGs, we first removed the matrigel from MG-hBORGs by treating them with cell recovery solution (Corning) for 1 hr under agitation at 4°C, followed by three washes with 1x PBS. We proceeded to RNA extraction of the pellet as previously described in this methods section.

### Immunofluorescence staining

Deparaffinized sections from frontal cortex tissues and organoids were rehydrated by three washes of PBS and five washes of 0.5% bovine serum albumin (BSA) and circled with a Liquid Blocker Mini Pap Pen (Life Technologies). Sections were further permeabilized with 0.1% Triton-X-PBS for 15 min followed by blocking with 2% BSA for 1 h. Sections were incubated with primary antibodies against human Nrgn (1:1000) overnight at 4°C. Tissues were washed five times with 0.5% BSA in PBS and were further incubated with goat anti-mouse-IgG Alexa Flour 488 and anti-rabbit-Cy5 followed by five washes with 0.5% BSA in PBS, and the nuclei were stained with DAPI (1:1000).

Coverslips from SH-SY5Y cells were fixed in 4% paraformaldehyde for 15 min, permeabilized and stained as previously described [4]. Lastly, slides were mounted with Gelvatol mounting medium and images were taken using confocal microscope.

### Confocal microscopy and Image Analysis

Confocal imaging was carried out using a Z-stacking function on the Nikon A1inverted confocal microscope using 40X dry or 60X oil objective at 4X zoom. For every image, we used a step size of 0.3–0.5mm for coverslips and paraffin sections totalizing 20–30 steps to image the entire layer of cells. Images shown are representative of cultures generated from 3 independent experiments (4 independent images/coverslip). Maximum intensity Z-projections were generated and analyzed using FIJI ImageJ 2.14/ 1.54f (National Institutes of Health, USA).

### Statistical analysis

Statistical analyses were performed using GraphPad Prism v 9.5.1 for Mac OSX (GraphPad Software, La Jolla, California USA). Three independent experiments were performed for all cell biological assays, unless otherwise stated. Experimental results were presented as the mean ± SE (standard error) and the difference were evaluated using the Student’s t-test with Welch’s correction or Pearson correlation test. P < 0.05 was considered to indicate a statistically significant difference.

## RESULTS

### HIV-1 infection decreases Neurogranin (Nrgn) expression in human brain tissue both at the mRNA and protein levels.

**Figure 1:**
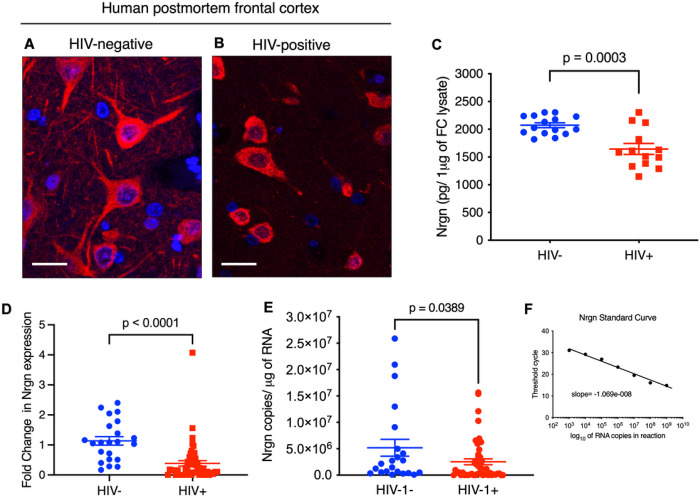
Neurogranin (Nrgn) is dysregulated in PWH at the mRNA and protein levels. Representative images of postmortem frontal cortex (FC) neurons stained for Nrgn (Cy3, red) and DAPI (blue) in **(A**) control and (**B**) people with HIV-1. The images are z-projections of image stacks acquired at ×60 magnification; scale bar is 10 mm. (**C**) Quantification of Nrgn levels in whole frontal cortex lysates from people with HIV-1 (N=13) in comparison with control individuals (N=15). (**D**) Relative expression of Nrgn mRNA in FC brain samples from HIV-1 positive individuals (N=49) compared to control individuals (N=22) as assessed by RT-qPCR. (**E**) Quantification of Nrgn mRNA copy number in FC samples of HIV-1 positive individuals compared to control individuals. (**F**) A standard curve obtained from serial dilutions of full-length Nrgn construct as template for the qPCR reaction to calculate copy number.

Simplification of dendritic network and synaptic dysfunction are neuropathological hallmarks of early HAND [3, 27, 28]. Previous report from our group indicated that post-synaptic protein, Nrgn is dysregulated in HAND positive individuals [4]. We then sought to evaluate the correlation between Nrgn expression and HIV-1 infection in the human brain cortex. Using immunofluorescence, we observed a dramatic decrease in Nrgn expression in frontal cortex tissue of HIV-positive individuals compared to healthy control brain tissues (Fig. 1A–B). We further quantitated the Nrgn level in whole frontal cortex tissue lysates in PWH (HIV-1+) (N = 13 donors) and age-matched people without HIV (HIV-) (N = 15) by Nrgn ELISA. We observed an average of 20% reduction in Nrgn level in HIV-1-positive individuals (Fig. 1C), compared to HIV-1 negative controls. Next, we assessed the relative expression level of Nrgn mRNA in total RNA samples from frontal cortex of HIV-1-positive individuals (N = 22) and age-matched control uninfected brain samples (N = 49) by RT-qPCR. Our results indicate that relative Nrgn mRNA level is significantly lower (average of 2.5-fold) in PWH in 75.5% of cases (37 out of 49) (Fig. 1D). Further assessment of copy number of Nrgn mRNA in brain tissue indicate that Nrgn is significantly reduced by 2.1-fold in HIV-1 positive individuals compared to HIV-1 negative individuals (Fig. 1E) based on the curve (Fig. 1F), confirming that decreased level of Nrgn protein in frontal cortex of PWH is associated with reduced amount of Nrgn mRNA.

### The candidate Nrgn regulator, lncRNA RP11–677M14.2 transcript expression is elevated in brain of HIV-1 positive individuals.

To identify the putative lncRNAs and its potential role in neurogranin dysregulation, we utilized the University of California Santa Cruz (UCSC) Genome Browser (genome.ucsc.edu) to investigate the genomic landscape of human NRGN gene. We have identified a single transcript (RP11–677M14.2) of 1,704 base pair length, which is localized in the antisense strand in NRGN locus in chromosome 11 (-strand, hg38) that remains to be characterized (**Additional file 1, Fig S1A, red arrow)**. Moreover, in Ensembl (http://www.ensembl.org) browser, this transcript corresponds to a 3 exons antisense RNA with no protein-coding potential, being classified as a long non-coding RNA. RP11–677M14.2 arises from independent promoter and its promoter region co-aligns to epigenetic markers of active transcription **(Additional file 1, Fig S1B, red circle, H3K27Ac mark)**. Analysis of the promoter activity from the same cell lines (**Additional file 1. Fig S1C, Regulatory build track, thick red blocks)** corroborates to this analysis, showing that when the Nrgn promoter is inactive (grey boxes on left), the RP11–677M14.2 promoter is active (red blocks on right). Additionally, gene expression data in 53 tissues from GTEx RNA-seq track revealed that RP11–677M14.2 is particularly abundant in human brain regions where Nrgn is also abundant, such as hippocampus and frontal **(Additional file 1, Fig S1D, yellow track)**[29]. Together, these data suggest a potential functional role for RP11–677M14.2 in mediating Nrgn levels.

**Figure 2:**
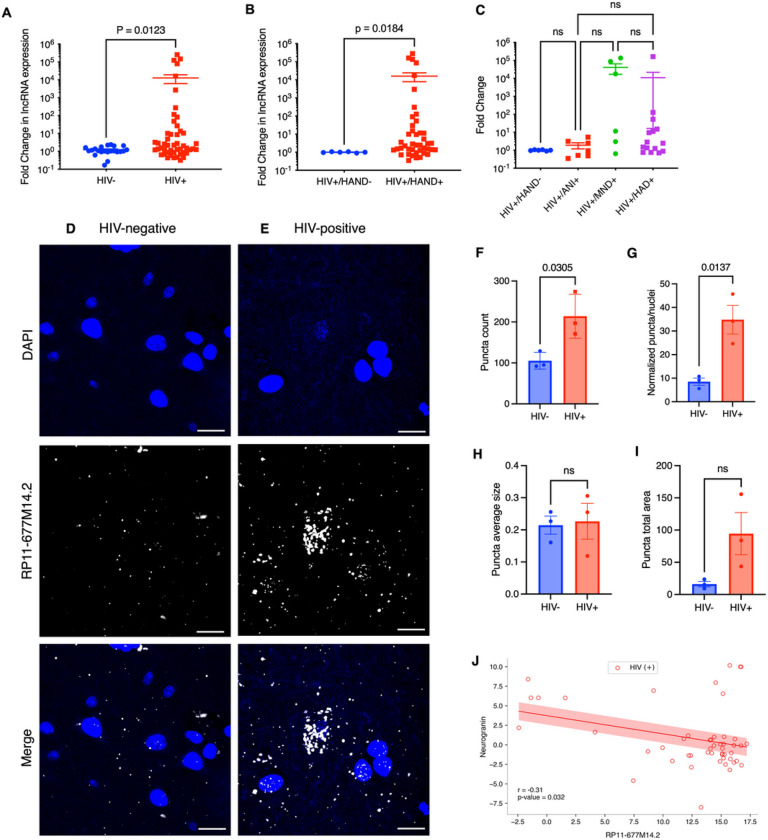
Expression analysis of RP11–677M14.2 transcript in frontal cortex tissues. (**A**) Relative expression of RP11–677M14.2 in FC from people with HIV-1 (N=49) compared with control individuals without HIV-1 (N=22) by RT-qPCR. (**B**) Relative expression of RP11–677M14.2 in HIV-1+/HAND-negative individuals (N=6) compared to HIV-1+/HAND-positive individuals (N=43). (**C**) Comparison of RP11–677M14.2 expression across different clinical stages of HAND. (**D-E**) Representative images of single molecule RNA HCR FISH with probe sets for RP11–677M14.2 transcript (AF-647, white puncta) in FC samples from people with and without HIV-1 (N= 3 per group). DAPI stain for cell nuclei is shown in blue. Scale bars show 10 μm. The images are z-projections of image stacks acquired at ×60 magnification. Quantification of the fluorescence signal from the experiment in panels D-E. For each the HIV-negative and HIV-positive data set, we quantified (**F**) total puncta count per image, (**G**) total puncta count normalized per cell number, (**H**) puncta average size and (**I**) puncta fluorescence signal intensity from averaged 5 images per individual per condition. (**J**) Pearson’s correlation analysis between the normalized expression of Nrgn mRNA and the normalized expression of RP11–677M14.2 in frontal cortex assessed by RT-qPCR (R=−0.31, P=0.032)

To investigate whether RP11–677M14.2 is dysregulated in HAND, we measured the lncRNA expression levels in frontal cortex tissues from age and sex-matched people with and without HIV by RT-qPCR assay. The levels of RP11–677M14.2 were aberrantly up-regulated (> 12.00 average fold-change, p = 0.0123) in 61.2% (30 of 49) of PWH compared with tissues without HIV-1 (Fig. 2A). Comparison between PWH without any degree of cognitive impairment (HIV+/HAND−) with PWH diagnosed with some level of cognitive impairment (HIV+/HAND+) also revealed a statistically significant increase in RP11–677M14.2 levels (Fig. 2B). Moreover, the relationship between RP11–677M14.2 expression and clinical stages of HAND was analyzed. Although not statistically significant, there is an upward trend of increased RP11–677M14.2 levels as HAND progresses from the less severe form (asymptomatic neurocognitive impairment, ANI) to the most severe form of disease (HIV-1-associated dementia, HAD) (Fig. 2C). To further confirm this expression pattern, next, we performed RNA-FISH HCR for RP11–677M14.2 transcript and co-stained with DAPI in 3 paired HIV-positive and HIV-negative FFPE autopsied human frontal cortex tissues. As shown in Figs. 2D and 2E, the lncRNA RP11–677M14.2 transcript is overexpressed in the brain tissue of PWH and the subcellular localization was predominantly in the cytoplasm. Calculation of total puncta counts (Fig. 2F) and the normalized puncta counts (Fig. 2G) further confirm that this lncRNA is globally upregulated in HIV-1 positive brains. Although no significant difference in puncta size and total area was observed between the two groups (Fig. 2H and 2I, respectively), we observed an increased lncRNA RP11–677M14.2 puncta area in brains of PWH (Fig. 2I). Further, the relationship between lncRNA RP11–677M14.2 expression level and Nrgn mRNA in 49 brain tissue samples of PWH was examined. Regression analysis showed that the overexpression of lncRNA RP11–677M14.2 was significantly correlated with lower levels of Nrgn in frontal cortex with a correlation coefficient of −0.3065 (p = 0.0322) as shown in Fig. 2J. Thus, we refer RP11–677M14.2 transcript as Nrgn antisense (Nrgn-AS).

### LncRNA RP11–677M14.2 displays both nuclear and cytoplasmic distribution.

**Figure 3:**
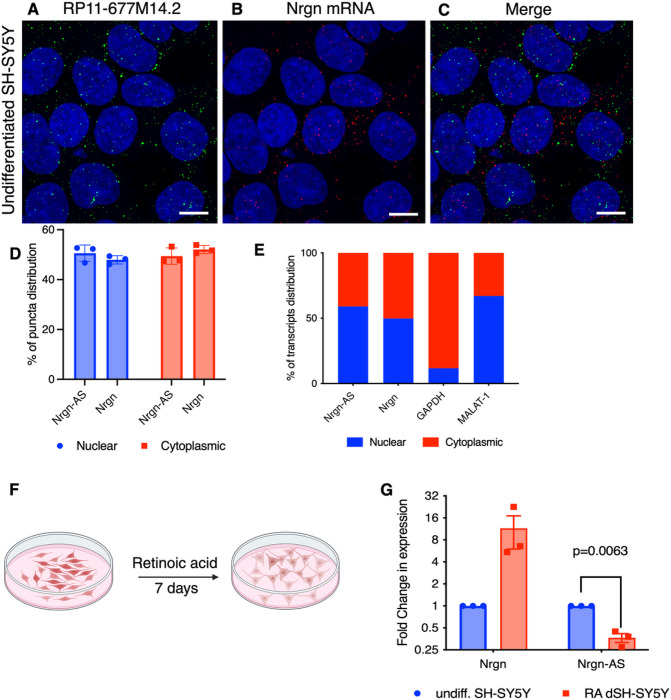
Transcriptional activation of Nrgn is associated with repression of RP11–677M14.2 lncRNA. Representative images of single molecule RNA multiplex HCR FISH with probe sets for (**A**) RP11–677M14.2 transcript (AF-488, green puncta) and (**B**) Nrgn mRNA (AF-647, red puncta) and (**C**) the colocalization of both transcripts in undifferentiated SH-SY5Y neuroblastoma cells. DAPI stain for cell nuclei is shown in blue. Scale bars show 10 μm, N= 3. The images are z-projections of image stacks acquired at ×60 magnification. (**D**) Distribution of RP11–677M14.2 transcript and Nrgn mRNA were quantified in different cellular compartments and presented as average percentage rate of total RNA puncta (N=3). (**E**) Relative RP11–677M14.2 and Nrgn mRNA levels in cytoplasm or nucleus of SH-SY5Y were detected by RT-qPCR. GAPDH was used as cytoplasm control and Malat1 was used as nucleus control. (**F**) Schematic of SH-SY5Y differentiation with retinoic acid (RA) treatment. (**G**) Assessment of Nrgn mRNA and RP11–677M14.2 transcript through RT-qPCR before and after SH-SY5Y differentiation treatment. N= 3 independent experiments.

LncRNAs have been separated into several broad classes in terms of their mechanisms of regulation of mRNA transcription and translation: decoys, regulators of translation, enhancers and modular scaffolds that guide chromatin modifying enzymes to specific genomic loci[30, 31]. Those lncRNAs localized within the nucleus, have been previously linked to the epigenetic control of transcriptional regulation through different mechanisms, whereas cytoplasmic lncRNAs are involved essentially in post-transcriptional mechanisms, subcellular localization and regulation of translation[32]. To dissect the function of Nrgn-AS in Nrgn regulation we first examined the distribution of this lncRNA (Fig. 3A) and Nrgn mRNA (Fig. 3B) in SH-SY5Y cells through RNA-FISH HCR co-stained with DAPI (Fig. 3C). Calculation of the puncta revealed that nearly 51% of the lncRNA transcripts reside in the nuclear compartment, whereas 48% of the Nrgn mRNA transcripts are localized within the nucleus (Fig. 3D)

Cell fractionation followed by RT-qPCR further confirmed that both RP11–677M14.2 transcript Nrgn mRNA are equally distributed between these two subcellular compartments (Fig. 3E). According to the distribution of glyceraldehyde-3-phosphate dehydrogenase (GAPDH) and Malat1, a lncRNA enriched in nucleus, the nucleus/cytoplasm separation was successful (Fig. 3E). Notably, minimal colocalization between the two transcripts was observed, suggesting that these transcripts do not physically interact (Fig. 3C)

### Nrgn and lncRNA RP11–677M14.2 (Nrgn-AS) transcripts exhibit discordant expression pattern.

Antisense transcripts may regulate expression of its sense gene at the transcriptional level (via transcriptional interference) and/or at the post-transcriptional level[7, 8, 33]. To investigate the potential regulation of Nrgn by endogenous levels of RP11–677M14.2, we treated SH-SY5Y cells with all-trans retinoic acid (RA) for 7 days (Fig. 3F) and measured the Nrgn and RP11–677M14.2 transcript by RT-qPCR. These cells express low levels of endogenous RP11–677M14.2 and high levels of Nrgn under normal conditions. During the differentiation process with RA treatment, both Nrgn mRNA and protein increase (Fig. 3G) Interestingly, our data reveal that while the expression of Nrgn increased, RP11–677M14.2 level sharply declined by 3-fold upon differentiation (Fig. 3B). This observation suggests that Nrgn level may be regulated by its anti-sense lncRNA RP11–677M14.2 in a discordant manner upon certain stimuli, as shown for other sense-antisense pairs[19, 34]. Therefore, we speculate whether this antisense lncRNA exerts a silencing effect on the Nrgn mRNA or corresponding protein abundance.

### Overexpression of Nrgn-AS, RP11–677M14.2 inhibits Neurogranin expression.

**Figure 4:**
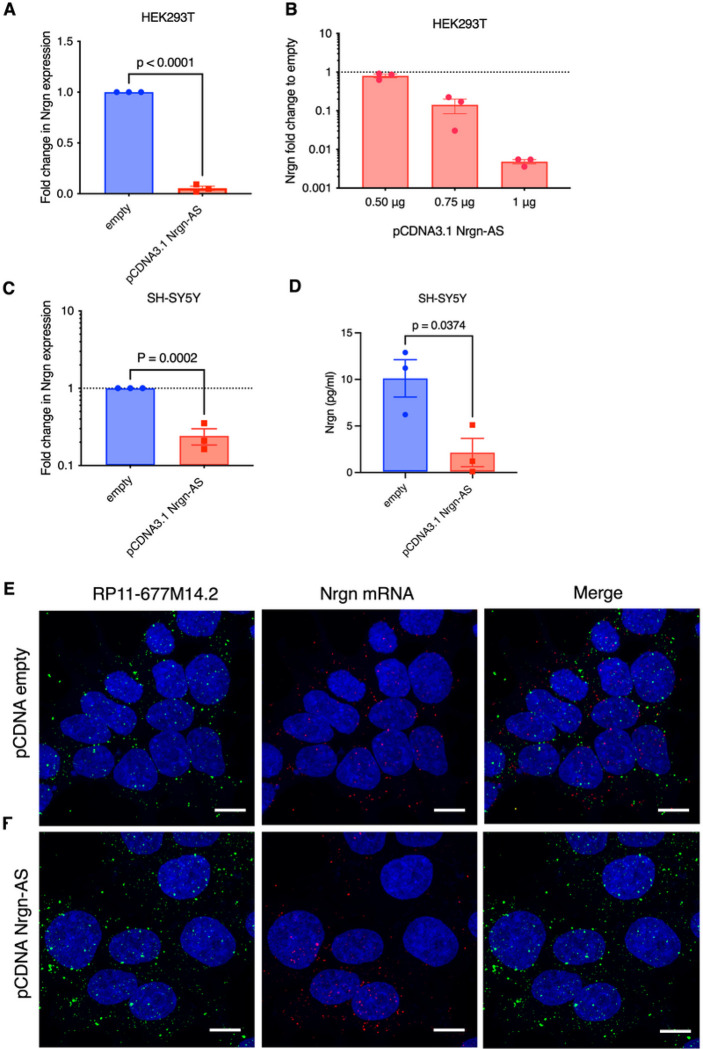
Overexpression of RP11–677M14.2 suppress Nrgn expression. (**A**) HEK293T cells were transiently transfected with pCDNA3.1 RP11–677M14.2 (pCDNA 3.1 Nrgn-AS) and after 48 hrs the levels of Nrgn mRNA were assessed by RT-qPCR in comparison with cells transfected with empty vector. (**B**) HEK293T cells were transfected with different doses of pCDNA3.1 Nrgn-AS as indicated, and levels of Nrgn mRNA were assessed after 48 hrs by RT-qPCR and compared with empty vector. Levels of (**C**) Nrgn mRNA and (**D**) protein were assessed by RT-qPCR and Nrgn ELISA respectively upon stably transfection of SH-SY5Y with pCDNA3.1 Nrgn-AS compared to empty plasmid (N=3). (**E-F**) Representative images of single molecule RNA multiplex HCR FISH with probe sets for RP11–677M14.2 transcript (AF-488, green puncta) and Nrgn mRNA (AF-647, red puncta) and the colocalization of both transcripts in undifferentiated stably transduced SH-SY5Y cells compared to SH-SY5Y transduced with empty vector. DAPI stain for cell nuclei is shown in blue. Scale bars show 10 μm, N= 3.

To test the prediction of a discordant regulation of Nrgn-AS and Nrgn mRNA, we have cloned full-length RP11–677M14.2 transcript into pCDNA3.1 expression vector to overexpress this lncRNA in HEK293T and SH-SY5Y cells **(Additional file 1, Fig S2A**). Firstly, Nrgn mRNA was assessed by RT-qPCR upon transiently transfecting the Nrgn-AS construct in HEK293T. Our results show that Nrgn mRNA was decreased by 90% in HEK293T cells in comparison to cells transfected with the empty vector (Fig. 4A). The effect seemed to be dose dependent as we observed concentration dependent decrease in Nrgn mRNA expression as we increase the quantity of pCDNA3.1 RP11–677M14.2 in transfection (Fig. 4B).

Next, we stably transfected SH-SY5Y cells with RP11–677M14.2 plasmid and observed a 50% reduction in Nrgn mRNA in these neuronal cells (Fig. 4C). To confirm these mRNA results, we next assessed Nrgn at the protein level by ELISA (Fig. 4D). Similar results were obtained when we measured the protein level of Nrgn, being observed an average decrease of 4.7-fold in neuronal cells overexpressing Nrgn-AS in comparison to cells transfected with empty vector as control. Finally, by employing FISH HCR co-stained with DAPI, we observed a diffuse distribution of the lncRNA in the stably transfected SHSY-5Y cells (Fig. 4F) similar to the endogenous distribution of this transcript observed in the empty vector control (Fig. 4E). These results suggest that the expression of Nrgn-AS directly or indirectly alters Nrgn mRNA expression resulting in lower protein levels.

To further investigate the effects of Nrgn-AS on the synaptodendritic damage, we also examined the expression level of selected synaptodendritic integrity markers: the dendritic marker MAP-2, the pre-synaptic markers GAP43, Synapsin and SNAP25, and the post-synaptic proteins Calmodulin, CAMK2, and calcineurin (PP3CA) in differentiated stably transfected SH-SY5Y (**Additional file 1, Fig S2B**). We observed a significant decreased expression of MAP-2 (p = 0.016377), SNAP25 (p = 0.0128) and CAMK2 (p = 0.003930) indicating that inhibition of Nrgn expression induced by overexpression of the lncRNA caused disruption of synaptodendritic integrity, which is implicated in cognitive decline.

### Supernatant from HIV-1 infected macrophages or microglia alters Nrgn-AS expression in neurons:

**Figure 5:**
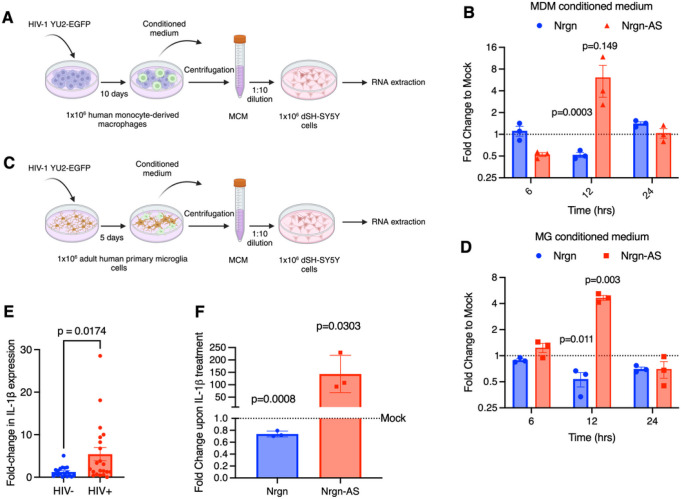
HIV-1 infection dysregulates Nrgn-lncRNA axis *in vitro*. **(A-D)** Schematic depicts experimental designed in which Nrgn-lncRNA expression axis was investigated: SH-SY5Y cells were differentiated with RA for 7 days and exposed to supernatant from HIV-1-infected or mock-infected (**A-B**) MDM or (**C-D**) Microglia. RNA was harvested at, 6, 12 and 24hrs of incubation and both the RP11–677M14.2 transcript and Nrgn mRNA levels were assessed by RT-PCR. (**E**) Relative expression of IL-1b in FC brain samples from HIV-1 positive individuals (N=21) compared to control individuals (N=18) assessed by RT-qPCR. (**F**) SH-SY5Y cells were differentiated with RA for 7 days and exposed to 0.1 ng of recombinant IL-1b for 1 hr and RNA was harvested. Nrgn and RP11–677M14.2 transcript expression level was assessed and compared to mock-treated neurons through RT-qPCR. Dotted lines represent the mock-treated neurons expression, blue bars represent average Nrgn fold change in expression whereas red bars represent the average lncRNA fold change in expression.

We next investigated whether HIV-1 infection affects Nrgn-AS transcript levels as it affects Nrgn mRNA levels *in vitro* by exposing differentiated SH-SY5Y cells to conditioned medium from HIV-infected monocytes-derived macrophages (MDM) (Fig. 5A–B), microglia cells (Fig. 5C–D) or mock-infected, to mimic the impact of HIV-1 induced inflammatory factors, toxins and/or viral proteins as previously described [35–37]. The effect of these factors on sense and antisense transcripts levels was assessed by RT-qPCR. Results indicate that exposure of cells to supernatant of HIV-infected MDMs resulted in an average 1.8-fold decrease in Nrgn mRNA and a 6.8-fold increase of Nrgn-AS levels (Fig. 5B), although the increase in the lncRNA expression varied between monocytes donors, it is not statistically significant (p = 0.149). Interestingly, our results show that there is a pattern of both transcripts being altered at the same time (between 6 and 12h post-exposure) and these alterations seem to be reversible as the stress factors degrade in the culture media (~ 24h post-exposure). Similarly, we tested the conditioned media obtained from HIV-infected human microglia and observed that exposure of cells to supernatant of HIV-infected microglia resulted in a 1.86-fold decrease in Nrgn mRNA and a 4.6-fold increase in Nrgn-AS levels (Fig. 5D) Together, these results suggest that both viral proteins and/or inflammatory factors released by infected MDM or microglia affect antisense transcript level. The composition of products released from infected MDM or microglia is complex and not fully known, however previous studies by us and others have identified several cytokines/chemokines that are known to have a role in neuropathogenesis [25, 35, 36]. Thus, to identify the proinflammatory cytokines present in HIV-1 infected MDM and microglia supernatants that might contribute to loss of Nrgn in neurons, we selected and measured the levels of interleukin IL-1b, IL-6, tumor necrosis factor TNF-a, and IL-8 in the supernatants of HIV-1 and mock-infected MDM (**Additional file 1, Fig S3A**) and microglia (**Additional file 1, Fig S3B**) by ELISA. As expected, HIV-1 infection increased the production and release of proinflammatory cytokines in both cell types. Among the pro-inflammatory cytokines tested, only IL-1b significantly increased upon HIV-1 infection in both MDM (p = 0.0210) and microglia (p = 0.0356) compared to mock infected. Next, we tested the levels of IL-1b mRNA in the frontal cortex tissues and observed a significant increase in IL-1b expression level in HIV-positive group (p = 0.0174) (Fig. 5E), but not TNF-a (p = 0.7487) (**Additional file 1, Fig S3C**), corroborating with these *in vitro* findings. Collectively, our results suggest that IL-1b released by infected MDM or microglia might in part mediate Nrgn-Nrgn-AS dysregulation in neurons.

To further test this hypothesis *in vitro*, we exposed differentiated SH-SY5Y to recombinant IL-1b (1mg/mL) for 1hr and assessed the levels of Nrgn and Nrgn-AS through RT-qPCR. Results indicate similar dysregulation of the sense-antisense axis in which Nrgn-AS, RP11–677M14.2 levels aberrantly increased by 143-fold, whereas Nrgn-mRNA levels decreased by 1.4-fold (Fig. 5F). This suggests that activation of an intracellular cascade downstream to IL-1b leads to transcriptional activation of Nrgn-AS which in turn downregulates Nrgn mRNA.

### Human brain organoids carrying HIV-1 infected microglia recapitulate the Nrgn-Nrgn-AS dysregulation.

**Figure 6:**
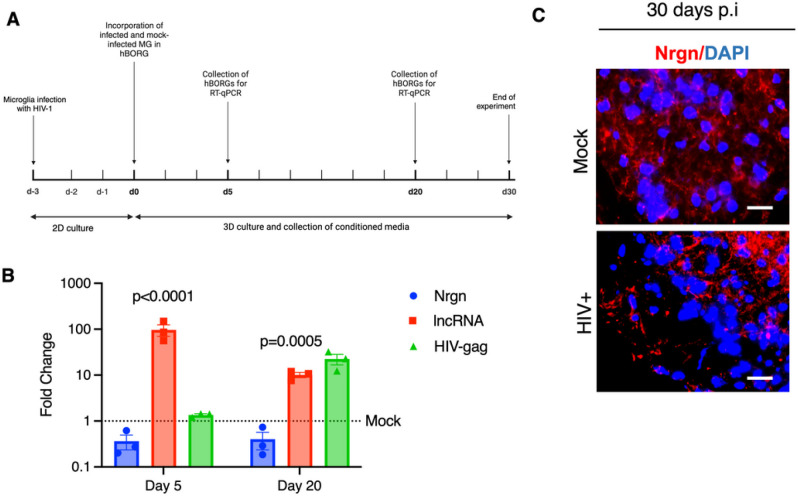
HIV-1 infection causes Nrgn dysregulation in Brain organoids. (**A**) Schematic diagram of the experimental design is depicted. Primary adult brain microglia (0.5 × 10^6^ cells) were infected with HIV-1 or mock-infected and were added to brain organoids for overnight. Microglia-embedded organoids were harvested at days 5 and 20 p.i. for RNA extraction, and at days 10 and 30 p.i. for immunostaining. (**B**) Mean of fold change variation in Nrgn, RP11–677M14.2 transcript and HIV-1 Gag expression compared to mock assessed through RT-qPCR (N=3). Dotted lines represent the expression levels of mock-infected brain organoids. (**C**) Immunostaining of infected brain organoid for Nrgn (Cy5, red) and nuclei (blue) compared to mock-infected organoid. DAPI Scale bars show 10 μm, N= 3. The images are z-projections of image stacks acquired at ×40 magnification.

To study Nrgn-Nrgn-AS dysregulation in a more physiologically relevant model, we leveraged 3D brain organoid technology by incorporating infected microglia to better represent the HIV-1 infected brain microenvironment. Having previously established that the triculture brain organoid system is amenable to HIV-1 infection resulting in increased glial activation and neuroinflammation [26, 38], we used our model to discern the contribution of Nrgn-AS dysregulation to Nrgn levels. As depicted in the schematic (Fig. 6A), we infected primary adult brain microglia with HIV-1 YU2-EGFP (MOI of 0.5) and 3 days post infection, microglia were incorporated into the fully mature brain organoids to generate an immunocompetent brain organoid as described[26]. These organoids were cultured for up to 30 days, harvesting RNA at days 5 and 20 for expression analysis. We found that incorporation of HIV-1 infected microglia caused substantial decrease in Nrgn expression (2.7-fold, p = 0.0056), whereas Nrgn-AS expression increased significantly (96-fold, p < 0.001) as early as 5 days in infected microglia containing organoids compared to uninfected control organoids (Fig. 6B). The same dysregulation continued up to day 20 p.i. where Nrgn mRNA and proteins levels are continuously suppressed (−2.5-fold, p = 0.0005) and Nrgn-AS level is elevated by 10-fold (p = 0.005) (Fig. 6B C). In addition, the low expression of HIV-1 Gag at day 5 post incorporation suggests that the rapid Nrgn mRNA dysregulation occurred prior to active viral replication, and it was sustained thereafter corroborating with our hypothesis that the inflammatory environment is driving the Nrgn-Nrgn-AS axis dysregulation. Finally, we assessed the expression of Nrgn protein level by immunohistochemistry at day 30 post-microglia incorporation. As expected, HIV-1 infection led to a decrease of Nrgn immunostaining and accumulation of the remaining protein in the perinuclear region (Fig. 6C).

## DISCUSSION

There is a clear correlation between Nrgn level and cognitive functions in humans [39–41]. We have recently demonstrated that Nrgn is dysregulated in frontal cortex of people living with HIV-1 suggesting that Nrgn loss might be an early event in the onset of HIV-associated cognitive disorder (HAND) [1]. Currently, there is no effective treatment to alleviate or restore cognitive deficits in HAND. Therefore, it is important to elucidate the molecular mechanism(s) underlying Nrgn dysregulation that results in learning and memory impairment. Long non coding RNAs (lncRNAs) are emerging as key players in regulating important cellular functions and a large volume of studies have linked the aberrant lncRNAs expression to a diverse number of human diseases[42]. However, very few lncRNAs are linked to HIV-1 infection and virtually none to the development and/or progression of HIV-1 associated neurocognitive disorder (HAND) [15, 16]

Here we present a novel lncRNA antisense transcript (RP11–677M14.2), named as Nrgn-AS, located in chromosome 11, same as NRGN locus, which function has not been identified. Expression of this lncRNA is negatively correlated with Nrgn mRNA levels in frontal cortex of HIV-1 positive individuals, suggesting that this lncRNA might be of relevance to Nrgn dysregulation. Thus, based on the *in vivo* evidence of a discordant regulation of the expression of sense and anti-sense transcripts, which is further supported by *in silico* analyses, we hypothesize that Nrgn-AS functions to suppress Nrgn expression.

Supporting our hypothesis, overexpression of RP11–677M14.2 in SH-SY5Y cells, led to significant decrease in Nrgn mRNA expression. Remains to be investigated, however, if treatment to silence RP11–677M14.2 transcript would restore Nrgn level in the neurons. Indeed, a previous RNA Seq assessment of the differential expression of miRNAs and lncRNAs has shown decreased expression of RP11–677M14.2 transcript in amygdala samples of schizophrenia patients in comparison with samples from control patients, whereas the expression of Nrgn mRNA was aberrantly higher in schizophrenia samples compared to controls [43]. Results of this study further strengthen our hypothesis that the dysregulation of the Nrgn-Nrgn-AS axis has important biological and functional relevance. Moreover, it indicates that it might be possible to increase Nrgn expression by modulating its antisense expression. Ideally, an effective therapeutic agent should preferentially increase Nrgn levels without disturbing physiologically essential basal expression levels. Future transcriptome analysis of cells endogenously overexpressing RP11–677M14.2 and cells silencing RP11–677M14.2 expression will clarify how specific is this anti-sense regulatory mechanism.

Post-transcriptional regulation of Nrgn through overlapping sense and antisense transcripts during cerebral corticogenesis and synapse function in mice have been reported previously [44]. Interestingly, our results showing discordant expression levels of sense and antisense before and after SH-SY5Y differentiation, suggests that RP11–677M14.2 acts as a temporal regulator of Nrgn as lncRNA is abundant in neuroprogenitor cells but suppressed soon after the neuronal differentiation is initiated. It is possible to speculate that the role of RP11–677M14.2 transcript in regulating Nrgn during neuronal differentiation/maturation is conserved both in mice and humans.

Our results from RNA HCR-FISH further demonstrated that RP11–677M14.2 transcript is evenly distributed throughout the cells as several other lncRNAs identified recently [18, 45, 46]. Subcellular localization of lncRNAs generally indicates their putative physiological roles. LncRNAs accumulated in the nucleus primarily mediate gene transcriptional repression or activation[47], whereas, lncRNAs accumulated in the cytoplasm interacts directly and/or indirectly with mRNAs to either stabilize the transcript for translation or target it to degradation [48, 49]. Remains unresolved whether the RP11–677M14.2 transcript exerts multiple roles in different cellular compartments as previously shown [50]. Regardless, lncRNA transcripts localization is a dynamic process and all lncRNAs are transcribed in the chromatin and therefore presenting nuclear expression. Eventually the transcripts are exported to the cytoplasm, a dynamic that still can be modified upon different stimuli and/or conditions.

We also demonstrated that RP11–677M14.2 transcript is upregulated in response to treatment with conditioned medium from HIV-1 infected macrophages/microglia. In our experimental setting, we observed that Nrgn mRNA dysregulation was synchronously influenced by RP11–677M14.2 lncRNA expression. While we cannot be certain that the Nrgn dysregulation upon exposure to supernatant of HIV-1 infected glial cells is due only to the up-regulation of RP11–677M14.2 lncRNA expression, these results provide preliminary evidence for a role to RP11–677M14.2 transcript in HIV-1 neuropathogenesis. Moreover, these observations suggest that the inflammatory factors released by infected macrophages may be in part responsible for Nrgn dysregulation through its anti-sense transcript (RP11–677M14.2). Furthermore, these supernatants also contain virus particles that have not been separated or inactivated. Future experiments will tease out the individual contribution of inflammatory factors, virus particles and viral proteins. Although our study was limited to the brain, investigation of differential expression of RP11–677M14.2 in plasma and/or CSF may also have a utility as a new biomarker for HAND as recently demonstrated for other antisense lncRNA [51].

## CONCLUSIONS

In summary, we have employed cellular and molecular approaches to investigate the role of RP11–677M14.2 lncRNA in Nrgn dysregulation. We have demonstrated that upregulation of the lncRNA in HAND may be partially determinant to Nrgn loss in neurons with potential implications to the onset and progression of neuropathogenesis. Insights obtained from this study will further advance our understanding of the molecular mechanism(s) underlying HIV-neuropathogenesis and provide additional opportunities to develop therapeutic targets.

## Data Availability

The data used to support the findings of this study are included within the article and its supplementary material. Sequence of RP11–677M14.2 transcript is publicly available, and it was retrieved from Ensembl (https://www.ensembl.org), Transcript ENST00000531241 from gene ENST00000531241.1. Values for all data points in graphs are available from corresponding author, upon reasonable request.
